# Weak functional connectivity in the human fetal brain prior to preterm birth

**DOI:** 10.1038/srep39286

**Published:** 2017-01-09

**Authors:** Moriah E. Thomason, Dustin Scheinost, Janessa H. Manning, Lauren E. Grove, Jasmine Hect, Narcis Marshall, Edgar Hernandez-Andrade, Susan Berman, Athina Pappas, Lami Yeo, Sonia S. Hassan, R. Todd Constable, Laura R. Ment, Roberto Romero

**Affiliations:** 1 Merrill Palmer Skillman Institute for Child and Family Development, Wayne State University, Detroit, MI 48202, USA; 2 Department of Pediatrics, Wayne State University School of Medicine, Detroit, MI 48202, USA; 3 Perinatology Research Branch, NICHD/NIH/DHHS, Detroit, Michigan, and Bethesda, Maryland, USA; 4 Institute for Social Research, Survey Research Center, University of Michigan, Ann Arbor, MI, 48104, USA; 5 Department of Radiology & Biomedical Imaging, Yale School of Medicine, New Haven, CT 06520, USA; 6 Department of Obstetrics and Gynecology, Wayne State University School of Medicine, Detroit, MI 48202, USA; 7 Department of Neurosurgery, Yale School of Medicine, New Haven, CT 06520, USA; 8 Department of Pediatrics, Yale School of Medicine, New Haven, CT 06520, USA; 9 Department of Neurology, Yale School of Medicine, New Haven, CT 06520, USA; 10 Center for Molecular Medicine, Wayne State University, Detroit, MI 48202, USA; 11 Department of Obstetrics and Gynecology, University of Michigan School of Medicine, Ann Arbor, MI, 48104, USA; 12 Department of Epidemiology, Michigan State University, East Lansing, MI 48825, USA.

## Abstract

It has been suggested that neurological problems more frequent in those born preterm are expressed prior to birth, but owing to technical limitations, this has been difficult to test in humans. We applied novel fetal resting-state functional MRI to measure brain function in 32 human fetuses *in utero* and found that systems-level neural functional connectivity was diminished in fetuses that would subsequently be born preterm. Neural connectivity was reduced in a left-hemisphere pre-language region, and the degree to which connectivity of this left language region extended to right-hemisphere homologs was positively associated with the time elapsed between fMRI assessment and delivery. These results provide the first evidence that altered functional connectivity in the preterm brain is identifiable before birth. They suggest that neurodevelopmental disorders associated with preterm birth may result from neurological insults that begin *in utero.*

Developmental problems are significantly more common in children born preterm. Epidemiological and meta-analytic studies indicate children born preterm are three times more likely to develop autism, attention deficit/hyperactivity disorders, and emotional disorders^1^,^2^, five times more likely to manifest neurological abnormalities^3^, and three to four times more likely to experience school failure^4^. Studies of the neuropathology of brain injury in the premature infant have identified alterations in cerebral white matter, grey matter, and projection fibers of the brain in infants born preterm^5^,^6^. Neural irregularities have been observed at the systems level as well, with altered wide-scale network connectional architecture identified in infants born preterm^7^,^8^,^9^,^10^. These studies report diminished coherence of activity measured across brain circuits, indicative of weaker connectivity in individuals born preterm from infancy^11^,^12^ through adulthood^13^,^14^. Given the robust linkage between child neurological disorders and atypical large-scale neural connectivity^15^,^16^ (see also refs 17, 18, 19, 20, 21, 22, 23, 24, 25, 26, 27, 28, 29, 30, 31, 32, 33, 34, 35, 36, 37), theory has evolved that neurodevelopmental impairment following preterm birth may stem from alterations in neural connectivity^38^, and recent evidence provides nascent support for this claim^10^,^39^.

A critical target for current research is to separate intrauterine from extrauterine influences on injurious brain development, and to isolate the earliest indicators of change in connectional architecture of the preterm brain. While much has been gained from examination of the postnatal preterm brain, examinations of human brain networks at or after preterm birth are confounded by potential insults conferred both by the absence of neuroprotective elements^40^,^41^ and addition of neurotoxic influences, which are inherent conditions of early delivery^42^,^43^. There has been an emphasis on hypoxia-ischemia and infection/inflammation as upstream etiologies of preterm brain injury, but there are several less frequently considered factors that also influence brain development, including extrauterine respiratory complications and the effects of fetal deprivation from maternal hormones and nutritional factors^41^. Studies of functional neural connectivity prior to preterm birth are needed to isolate processes that begin in the womb. If functional connectivity is altered in the preterm brain *in utero*, the untoward influences of extrauterine factors cannot be the source of those differences. The recent development of resting-state fMRI (rs-fMRI) methodology for the human fetus offers the first opportunity to investigate altered functional connectivity prior to birth^44^,^45^.

In this study we utilized rs-fMRI to measure neural connectivity *in utero* in 32 human fetuses, 14 of which were subsequently born preterm. We employed an intrinsic connectivity distribution analysis of rs-fMRI data to map synchrony in MRI signals over time across the fetal brain. We then investigated the relationship between connectivity and gestational age at fMRI assessment and delivery. Lastly, post-hoc seed analysis was performed to explore the regions in which specific connections were most responsible for changes intrinsic connectivity distribution (ICD)^46^ value differences between preterm and full-term groups.

## Results

### Description of research cohort

Thirty-six women, mean age 25.3 years, SD, 5.6, underwent MRI between their 22nd and 36th week of pregnancy. Half of participating pregnant women were high-risk for early delivery and gave birth prior to the 37th week of pregnancy, mean GA preterm birth = 32 weeks, range = 24–35 weeks. The comparison group was case control matched based on GA at time of MRI and gender of the fetus (Table 1). Four high-risk participants were excluded due to fetal intrauterine growth restriction, or IUGR, which can influence neural network connectivity^47^. The final study sample consisted of 14 pregnancies that ended in preterm delivery between 24 and 35 weeks, and 18 uncomplicated pregnancies. Birth and placental pathology outcomes of the preterm sample are provided in Supplemental Table 1.

Studies that demonstrate postnatal differences in brain development as a function of sex^48^, maternal prenatal stress^49^, and socioeconomic status^50^ introduce the possibility that intrauterine functional connectivity may differ as a result of such factors. We examined the possibility that these or other key indicators may differ between our study groups. No differences were observed between study groups in fetal age or gender, maternal demographic or IQ measures, nor fMRI data quality measures (Table 1). From this we infer that group differences do not result from features of the social and psychological context that may differ between groups or from data quality, but rather the altered brain development in fetuses later born preterm.

### Diminished connectivity in fetuses later born preterm

**Figure 1:**
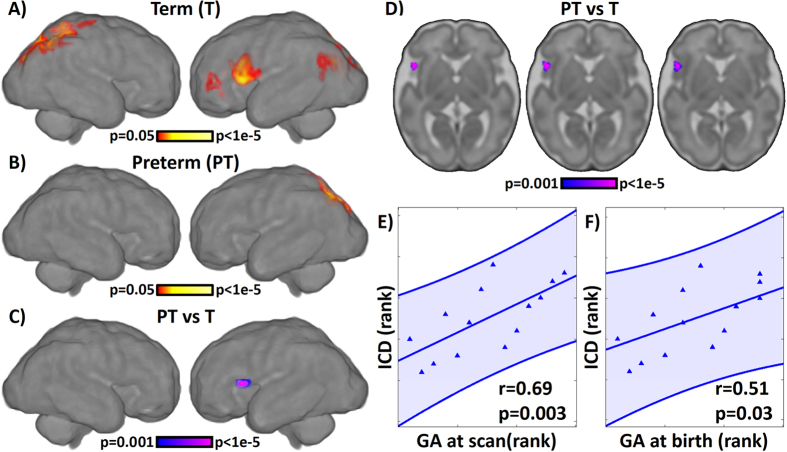
Comparison of preterm (PT) - and term (T) -born fetuses using voxel-level connectivity. Single group maps for (**A**) term and (**B**) preterm-born fetuses, showing putative hub regions in the fetal brain at 29.6 weeks. Significant differences (p < 0.05, corrected) between preterm- and term-born fetuses were observed in eventual left hemisphere language regions in the frontal lobe as shown on (**C**) surface rendering, and (**D**) axial slices. No regions exhibited increased ICD for preterm-born fetuses compared to term born fetuses. For the preterm-born fetuses, ICD values extracted from a seed centered on the peak difference (27 mm left, 18 mmanterior, 5 mm inferior of the center of image data) were significantly correlated with both gestational age (GA; panel (**E**)) at scan (r = 0.69, p = 0.003, df = 12) and (**F**) at birth (r = 0.51, p = 0.03, df = 12) using one-tailed Spearman rank correlation.

ICD comparisons of preterm- and term-born fetuses revealed a substantial area of the left hemisphere, proximal to what will later become Broca’s area, where connectivity was greater in term-born fetuses (Fig. 1). In contrast, there were no areas in which connectivity was greater in those later born preterm. Observed effects agree with evidence of atypical brain connectivity in preterm born neonates obtained using similar MRI systems level analyses^11^,^12^,^51^, and animal electrophysiological and cellular models of prematurity and inflammation that report diminished dendritic branching, arborization, oligodendrocyte maturation, and cell-to-cell communication in affected animals^52^. A follow up analysis examined the possibility that preterm labor unduly contributed to group differences, and found that even after removing 4 fetuses born within 7 days of the scan, the effect remained significant (Supplemental Fig. 1). This effect also remained significant when repeated in a multiple linear regression model with age, sex, and motion variables as covariates (Supplementary Table 2). Differences in connectivity in this putative pre-language region support the notion that language impairments and related altered connectivity often observed in individuals born preterm^53^,^54^,^55^ may arise from altered brain development that commences *in utero*.

### Neural connectivity relates to age at MRI and length of gestation

We reasoned that if this fetal pre-language region is a critical hub of connectional group differences, then variation in connectivity of this region may relate to individual differences in the preterm-born sample. Correspondence between functional connectivity, or ICD, of this region, and both gestational age at the time of scan and gestational age at delivery were evaluated. Generally, postnatal evaluation of the human premature brain has pointed to early developmental delays, some of which improve with age, either through restorative or compensatory processes^56^. We therefore expected that gestational age at time of scan may relate to the level of connectivity in this region, with older preterm-born fetuses demonstrating connectivity values more similar to the term-born controls. Results supported this idea, with positive correspondence between rank age at scan and values in the ICD peak region in the preterm group, r = 0.69, p = 0.003 (Fig. 1E). An analogous examination of the association between neural functional connectivity and gestational age at delivery revealed, again, a significant positive correlation in preterm fetuses within the ICD peak region, r = 0.51, p = 0.03 (Fig. 1F). This latter observation suggests that more significant impairments observed in those born extremely preterm may begin with altered neural functional connectivity before birth. Together, these findings demonstrate that the extent to which this pre-language region is connected with other brain regions, rather than functioning independently, is related to both gestational age at scan and length of gestation. Those destined to have longer gestational duration, were more similar in functional connectivity to those born at term.

### Altered proto-linguistic functional connectivity

**Figure 2:**
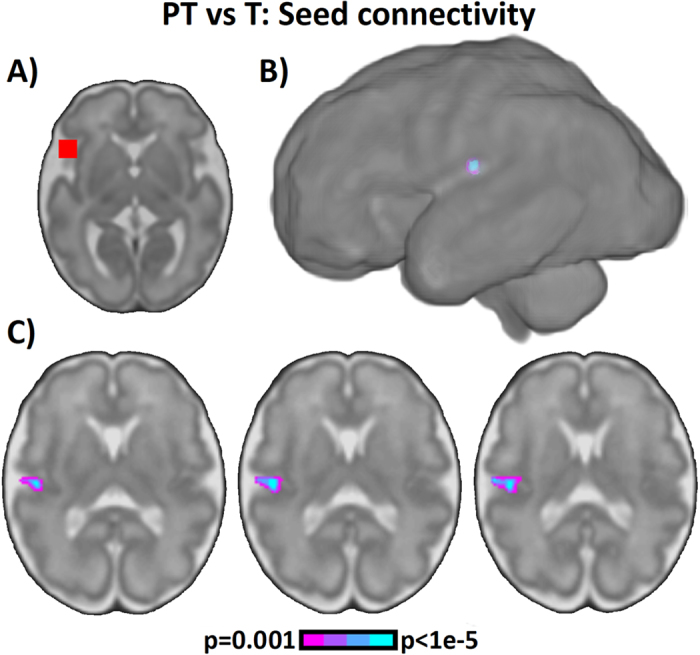
Comparison of preterm- and term-born fetuses using seed connectivity. (**A**) To investigate which specific connection may be driving our voxel-level ICD results, we performed follow-up seed connectivity using a cubic seed (shown in red) centered at the peak coordinate of between-group differences (27 mm left, 18 mm anterior, 5 mm inferior of the center of image data). (**B**) Significant differences (p < 0.05, corrected) between preterm- and term-born fetuses were observed in regions eventually becoming primary auditory cortex and Wernicke’s area as shown on (**B**) surface rendering and (**C**) axial slices. No regions exhibited increased seed connectivity for preterm-born fetuses compared to term-born fetuses.

To better understand differences between groups, we performed follow-up region of interest seed connectivity analysis using this putative pre-language, ICD peak region as an area of interest. Signal from this region was correlated with signal intensity in every other grey matter voxel to identify brain regions that demonstrated similar signal properties over time. In the preterm-born fetal group, we observed diminished signal correlation in ipsilateral posterior superior temporal gyrus, extending into the posterior pre-language brain network (Fig. 2; Supplementary Table 2), when compared to term-born control cases. Previously, we have shown in typically developing fetuses that a large decrease in modularity between these regions occurs during the third trimester^57^, indicative of increased crosstalk between these regions with advancing age. Our current results indicate that functional organization of the larger pre-language system, encompassing both Broca’s and Wernicke’s areas, may be reduced in fetuses who subsequently are born preterm.

### Remaining gestational duration linked to contralateral connectivity

**Figure 3:**
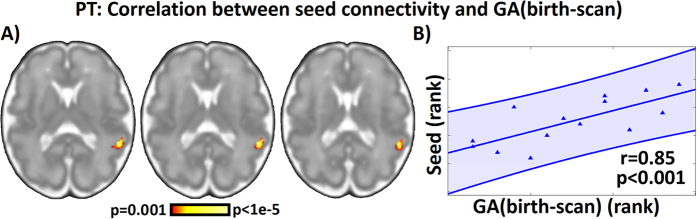
Whole brain regression of time between scan and birth for fetuses (n = 14) subsequently born preterm. Using the same seed connectivity as in Fig. 2, we investigated whether specific connections to the seed region were associated with increased time between fetal MRI and birth. (**A**) Connectivity between the left hemisphere proto-language seed and right hemisphere language homologues in the parietal lobes were significantly correlated (p < 0.05, corrected) with increased time between fetal MRI and birth. No regions exhibited a significant negative correlation. This association suggests that cross-hemisphere connectivity between language regions may be predictive of longer *in utero* development for those at risk of preterm birth. Connectivity values averaged from a seed centered on the peak difference and linear fit from spearman rank correlation are extracted for each subject and used to visualize the observed effect in (**B**). Using partial spearman correlation, these correlations remained significant after controlling for gestational age (GA) at scan (r = 0.78, 0 = 0.002, df = 11) and GA at birth (r = 0.83, p = 0.001, df = 11), suggesting a unique effect of time between fetal MRI and birth.

We also examined connectivity of the area of peak differences between groups, the anterior pre-language region, within the preterm fetal group to ascertain whether connectivity profiles were different in fetuses who would be born soon after their MRI date, versus those born further out from the time of MRI. Whole-brain regression from the peak ICD region revealed that connectivity to right-hemisphere pre-language homologues was diminished in those fetuses that would be born soon after MRI, defined as duration of time between scan and delivery. This effect remained significant even when controlling for age at scan and age at delivery (Fig. 3). This is striking given knowledge from our prior work that connectivity to contralateral hemisphere neural homologues increases with advancing fetal age^44^. This new observation suggests that fetuses at risk for impending preterm delivery had weaker left to right hemisphere connectivity in this lateral fronto-parietal region, the region that differentiated preterm versus term born whole-brain functional connectivity. This altered connectivity between hemispheres *in utero* may set the foundation for altered cross-hemisphere connectivity observed in infants^51^, children^55^, and adolescents^53^,^54^ born preterm.

### Underdetermined role of inflammation in altered neural functional connectivity

Conditions that increase risk for prematurity may also be those that enhance risk for deficient neural functional connectivity of the fetus. Intra-amniotic infection increases risk for both preterm delivery and white matter injury^58^,^59^. The association between intra-amniotic infection and neural connectivity is as yet untested in humans, but is indirectly supported by postnatal data demonstrating links between white matter injury and neural functional connectivity measured in premature infants^60^. Placental pathology reports obtained for 28 participants in our study sample were evaluated for presence or absence of inflammatory lesions. Pathology confirmed high prevalence of inflammatory lesions, either acute or chronic, manifested as focal or generalized acute chorioamnionitis, chronic chorioamnionitis, and funisitis in the study sample (Supplemental Table 1). Indicators of inflammation were present in placental histology reports of all women in the preterm group. As expected there were significantly fewer indications of inflammatory lesions in the comparison group, χ^2^(2, *N* = 28) = 8.089, *p* = 0.007. These clinical variables may relate to neural functional connectivity, but due to small sample size and potential for type II error, it was not appropriate to examine those potential associations here.

## Discussion

Defining the nascent architecture of the preterm brain provides a basis for understanding the underlying etiology of neurological impairments that can accompany prematurity. Using recent developments in fetal resting-state fMRI we examined neural functional connectivity in 32 human fetuses and found reduced connectional integrity in fetuses that would subsequently be born preterm, particularly in regions of the left hemisphere that later support language processing. Strength of functional connectivity in this region was related to gestational age at delivery, such that those born closer to expected due date demonstrated connectivity profiles more similar to those in the case-matched control group. Thus, observed differences in neural functional connectivity were related to proximal health outcomes in the preterm group.

These results demonstrate that neurological connectivity differences associated with human preterm birth begin *in utero*, prior to the potentially injurious experiences of early delivery. These constitute the first human data to suggest that disabilities frequently accompanying extreme prematurity, such as autism and ADHD, may derive from pre-existing intrauterine neurological conditions, especially given that these disorders have neuroconnectional bases^61^,^62^,^63^,^64^. We found that diminished functional connectivity was also linked to individual differences in the preterm group, suggesting that maturation of brain circuitry may be associated with the circumstances of an individual pregnancy. We observed that both gestational age at time of scan and gestational age at delivery positively relate to the degree of connectivity, such that fetuses with increased intrauterine course demonstrate connectivity values more similar to the term-born controls. This pattern builds on prior multi-level postnatal neuroscience findings demonstrating that brain circuits flexibly adjust across the life-span and in response to injury, environmental programming, and/or disease^65^,^66^. Similarly, fetuses born preterm often overcome initial functional deficits during postnatal or early childhood development. Our results indicated that left-hemisphere pre-lingual regions and cross-hemispheric connections were most significantly impacted, fitting with the language deficits that frequently manifest in those born extremely preterm^67^,^68^. This observation may also imply centrality of lingual brain networks in organization of emergent functional brain systems.

Knowledge that functional connectivity differs before birth in those born preterm encourages evaluation of prenatal intervention. Potential efficacy of prenatal neurobehavioral therapy is supported by growing evidence from non-human animals and from humans that learning occurs prior to birth. Markham and colleagues have demonstrated that birds learn about the calls of conspecifics while still in the egg, and that this embryonic programming alters neural cellular activation after birth and also characteristics of later vocalizations^69^. A landmark study by Partanen and colleagues presented evidence of experienced-based neuroplasticity in *human fetuses*. This group exposed fetuses to varied quantities of word-like sounds and found that, after birth, those exposed to intrauterine stimuli showed behavioral learning and enhanced brain activity that scaled with quantity of exposure^70^. They concluded that prenatal exposure to complex sounds may lead to the development of a more effective neural network for information processing after birth. These studies substantiate presence of cross-species learning-induced neural plasticity *in utero*. Understanding that extrauterine environmental stimuli not only induce behavioral response in the fetus, but also change brain function in lasting ways, supports the possibility that prenatal behavioral intervention may serve as an effective therapy for those at risk.

While this study presents a novel comparison of human brain function before birth in fetuses destined either for term or preterm delivery, considerations about nascent fetal fMRI methodology merit discussion. Challenges inherent in the methodology include small fetal head volume, influences of physiological signals originating from mother and fetus, limited constraints over motion, and variation in orientation^71^. These concerns are not entirely unique to *fetal* fMRI, as partial volume effects, physiological noise, motion, and image registration are broad concerns for the larger MR field, and many strategies exist for addressing these^72^,^73^,^74^,^75^, several having been applied in the present study. However, the fetus represents an extreme case for these crucial areas and considerable work remains to be done to bring fetal fMRI to its full potential, including such things as development of specialized tools and atlases for fetal MRI. Until that time, current best practices include normalization to age-appropriate fetal templates^76^, use of subject specific anatomical segmentation, and use of data-driven, rather than spatially constrained, analytic approaches such as independent components analyses^77^, spatial-spectral parcellation^57^,^78^, ICD^46^ (employed here), and multivariate distance-based analyses^79^ methods.

Another topic requiring careful consideration in fetal fMRI is that we possess limited understanding of the physiological basis of blood-oxygen level dependent (BOLD) fMRI signals in the fetal brain. Current understanding is derived from assumptions regarding neurovascular coupling that stem from neurophysiological research performed in animals^80^. However, emergent data suggests some of these assumptions may be justified even in the immature human fetal brain. For example, studies are beginning to show that fetal functional connectivity MRI measures are congruous with what we know about neural development during this time. We and others have shown that intrahemispheric^44^ and long-range^81^,^82^ fetal fMRI signals become more synchronized with age, which mirror known principles of fetal anatomical development^83^,^84^. In addition, the few studies that have investigated BOLD signal in the antenatal period report positive BOLD contrast responses in preterm infants^85^ and in animal models of the preterm period^86^. Furthermore, murine studies of pre- and postnatal angioarchitechtonics support tight coupling of neural and vascular dynamics across early development^87^. Overall, it is well reasoned that signal covariation measured in fetal resting-state studies reflects the establishment of communicative architecture of the brain. While support for this is scarce and interpretive caution is advisable, available data do support this position.

In conclusion, we provide the first evidence that neural pathways are likely to be altered prior to preterm delivery. This discovery suggests that factors influencing early delivery may also impact development of the human brain, which has implications for life-long health. Future work will address sources such as infection and inflammation that may play a causal role in altering these parallel pathways, bringing us closer to understanding both the primary neurological injury and the optimal timing for early intervention.

## Methods

### Participants

Singleton pregnancies with normal brain anatomy assessed by ultrasound and MRI examination reporting no contraindications for MRI were eligible to participate. MRI T1 weighted images revealed no brain injury in any fetal cases at the time of prenatal MRI examination. Despite that no areas suggestive of brain lesions were observed, the presence of micro bleeds, or other more subtle forms of injury, cannot be completely excluded. The mean age of fetuses at the time of MRI was 29.9 weeks, SD, 3.4, post-menstrual gestational age (GA). Ultrasound (US) examination administered by study physician (E.H.-A.) was performed within 1 week of MRI examination to determine fetal GA. All women were native English speakers. All women provided written informed consent before undergoing MRI examination. Participation was approved by the Institutional Review Board of the National Institute of Child Health and Human Development (NICHD) and by the Human Investigation Committee of Wayne State University. All experiments were performed in accordance with relevant guidelines and regulations.

### Image acquisition

Fetal MR exams were performed with a Siemens Verio 70-cm open-bore 3-T scanner with a light-weight (~550 g) abdominal 4-Channel Siemens Flex Coil. The MRI examination lasted 45 min. Images were collected with the following parameters and computed SAR values: (i) localizer [repetition time/echo time (TR/TE), 20/4.2 ms; 10-mm thickness; SAR = 0.21]; (ii) T2 anatomical (TR/TE, 3500/140 ms; 3-mm slice thickness; repeated 4–6 times; SAR = 0.53); (iii) echo planar imaging (EPI) BOLD (TR/TE, 2000/30 ms; 180 frames; 4-mm slice thickness; axial; repeated twice; SAR = 0.3); (iv) susceptibility weighted imaging (SWI) (TR/TE, 30/21.2 ms; 4-mm slice thickness; SAR = 0.06).

### Connectivity preprocessing

Fetal fMRI data were censored^75^ for motion using criterion <1 mm frame-to-frame translation and <1.5 degrees rotation. 56% of data collected were retained after motion censoring. Censored data were motion corrected, normalized to common template space, and smoothed as previously described^57^ using both manual and automatic methods. Further connectivity analysis was performed using BioImage Suite^88^. Several covariates of no interest were regressed from the data including linear and quadratic drifts, 6 motion parameters, mean cerebral-spinal-fluid (CSF) signal, and mean white-matter signal. The data were temporally smoothed with a zero mean unit variance Gaussian filter (approximate cutoff frequency = 0.12 Hz). A gray-matter mask defined in template space was applied to the data so only gray matter voxels were used in further calculations.

### Intrinsic functional connectivity

**Figure 4:**
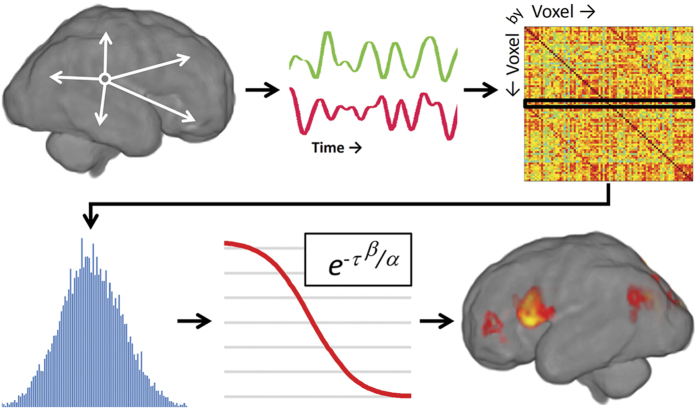
Overview of ICD analysis. For any voxel in the gray matter, the voxel’s time course is correlated with every other voxel’s time course. This procedure is repeated for every voxel, resulting in a voxel-by-voxel correlation matrix. From this matrix, a single row is extracted (representing all correlation to a voxel) and converted to a histogram to estimate the distribution of connectivity for that voxel. From this distribution, a survival function is constructed and parameterized with a stretch exponential with unknown parameters α and β. For our purposes, α controls the rate of decay of the survival function with a larger α indicating a slower decay and larger global connectivity. The α ICD maps can then be thresholded to reveal putative hubs of the fetal connectome.

After preprocessing, intrinsic connectivity distribution (ICD; Fig. 4) was computed at the voxel level for each subject as described previously^46^. Similar to most voxel-based functional connectivity measures, ICD involves correlating the time-course for any voxel with every other time-course in the brain or brain hemisphere, and then a summary statistic based on the network theory measure *degree* was calculated. ICD models the entire distribution of correlation thresholds using a Wiebull distribution avoiding the need for choosing an arbitrary connectivity threshold. This parameterization is akin to modeling the change in network theory metric *degree*, as the threshold used to calculate *degree* is increased, with a stretched exponential. Specifically, the time-course for any gray matter voxel was correlated with every other voxel in the gray matter. A histogram of these correlations was constructed to estimate the distribution of connections to the current voxel. This distribution was converted to a survival function and the survival function was fitted with a stretched exponential with unknown variance. As variance controls the spread of the distribution of connections, a larger variance indicates a greater number of high correlation connections. Finally, this process is repeated for all voxels in the gray matter resulting in a whole-brain parametric image summarizing the connectivity of each tissue element. ICD was computed in each study group (Supplemental Fig. 2) and compared to identify brain regions where functional connectivity differed between groups.

To interrogate relative differences in connectivity, each participant’s map was normalized by subtracting the mean across all voxels and dividing by the standard deviation across all voxels. This z-score-like normalization does not change the underlying connectivity pattern but allows for investigation of relative differences in connectivity in the presence of large global differences in connectivity^89^.

### Follow-up seed connectivity

Follow-up seed analysis was performed to explore (post-hoc) connectivity of the node where ICD connectivity differed most significantly between groups. Signal intensity was extracted from voxels centered on the ICD peak at location 27 mm left, 18 mm anterior, 5 mm inferior of the center of image data registered to a common 32-week fetal brain template^76^. The time course of the seed region in a given participant was then computed as the average time course across all voxels, comprising a 512 mm^3^ cube, in the seed region. This time course was correlated with the time course for every other voxel in the gray matter to create a map of r-values, reflecting seed-to-whole-brain connectivity. These r-values were transformed to z-values using Fisher’s transform yielding one map for each participant representing the strength of correlation to the seed region.

### Statistical analysis

For imaging data, voxel-wise two sample independent t-tests were used to compare the connectivity data between groups. Voxel-wise Pearson’s correlation was used to assess association between connectivity and GA. Spearman rank correlation and partial Spearman rank correlation were used to assess the association between extracted connectivity values and GA. Results are shown at a cluster-level threshold of p < 0.05 family-wise error (FWE) correction as determined by AFNI’s 3dClustSim program. The cluster threshold was determined using cluster-forming threshold of p = 0.001, 10,000 iteration, smoothness estimated using a mixture of Exponential and Gaussian distributions (i.e. the –acf option), and 16.0.09 release of AFNI. For non-imaging data, two sample independent t-tests and chi-square tests were used with significances assessed at p < 0.05.

## Additional Information

**How to cite this article**: Thomason, M. E. *et al*. Weak functional connectivity in the human fetal brain prior to preterm birth. *Sci. Rep.* **7**, 39286; doi: 10.1038/srep39286 (2017).

**Publisher's note:** Springer Nature remains neutral with regard to jurisdictional claims in published maps and institutional affiliations.

## Supplementary Material

Supplementary Information

## Figures and Tables

**Table 1:** Summary of participant and fMRI data characteristics by group.

	Fetuses (n = 14) later born prematurely	Fetuses (n = 18) later born at term
Fetal GA at scan	29.6 (3.7)	30.3 (3.2)
Fetal GA at delivery*	32.4 (4.2)	38.8 (1.1)
Maternal depressive symptoms, m (SD)	15.3 (12.1)	12.4 (8.5)
Maternal anxiety symptoms, m (SD)	36.9 (12.7)	34.1 (8.2)
Maternal stress, m (SD)		
Penn State Worry Questionnaire	19.9 (9.0)	14.9 (5.9)
Perceived Stress Scale*	46.1 (14.8)	40.2 (8.7)
Fetal gestational age at MRI, m (SD)	29.7 (3.7)	30.25 (3.3)
Maternal IQ, m (SD)	82.8 (13.8)	78.8 (13.1)
Ethnicity, n (%)		
Caucasian	0	1 (5.6)
African-American	13 (92.9)	14 (77.8)
Asian	0	1 (5.6)
Other	1 (7.1)	2 (11.1)
Education, n (%)		
No GED/High-school diploma	3 (21.4)	6 (33.3)
GED/High-school diploma	3 (21.4)	5 (27.8)
Some college	6 (42.9)	6 (33.3)
2-yr college degree	1 (7.1)	1 (5.6)
4-yr college degree	1 (7.1)	0
Annual Income, n (%)		
<$10,000	9 (64.3)	6 (35.5)
$10,000–$20,000	2 (14.3)	7 (41.2)
$20,000–$30,000	2 (14.3)	3 (17.6)
$30,000–$40,000	0	1 (5.9)
>$40 000	1 (7.1)	0
Motion during resting-state scan		
Translational mean movement, m (SD)	0.4 (0.1)	0.4 (0.1)
Rotational mean movement, m (SD)	0.7 (0.3)	0.7 (0.4)
Translational RMS, m (SD)	0.2 (0.1)	0.3 (0.1)
Rotational RMS, m (SD)	0.0 (0.0)	0.0 (0.0)
fMRI data characteristics		
SAR, m (SD)	0.26 (0.1)	0.3 (0.1)
Number of fMRI frames analyzed, m (SD)	171 (81.2)	185.4 (50.4)
Proportion frames retained after exclusion of periods of movement, % (SD)	0.6 (0.2)	0.5 (0.1)

Chi-square tests compared race/ethnicity, education, and income between groups. Two-sample independent t-tests compared all other variables. As planned, fetal GA at delivery* was significantly different, p < 001. All other comparisons were non-significant, using two-tailed p < 0.05. A single trend was observed for the Perceived Stress Scale** at p = 0.066; p’s > 0.1 for all other comparisons. Depressive and anxiety symptoms were measured using the Center for Epidemiologic Studies Depression Scale and State-Trait Anxiety Inventory, respectively. Intelligence Quotient (IQ) was measured using verbal and matrix reasoning subtests of the Wechsler Abbreviated Scale of Intelligence. Gestational age (GA) reported in weeks; translational (x, y, z) movement reported in mm; rotational, in degrees; Specific Absorbtion Rate (SAR) in units of watts per kilogram (W/kg). Abbreviations: standard deviation, SD; mean, m; root-mean-square, RMS (head position change).

## References

[b1] Bhutta A. T., Cleves M. A., Casey P. H., Cradock M. M. & Anand K. J. Cognitive and behavioral outcomes of school-aged children who were born preterm: a meta-analysis. Jama 288, 728–737 (2002). 12169077 10.1001/jama.288.6.728

[b2] Larsson H. J. . Risk factors for autism: perinatal factors, parental psychiatric history, and socioeconomic status. American journal of epidemiology 161, 916–925, discussion 926-918, doi: 10.1093/aje (2005). 15870155

[b3] Miller S. P. . Early brain injury in premature newborns detected with magnetic resonance imaging is associated with adverse early neurodevelopmental outcome. J Pediatr 147, 609–616, doi: 10.1016/j.jpeds (2005). 16291350

[b4] Buck G. M., Msall M. E., Schisterman E. F., Lyon N. R. & Rogers B. T. Extreme prematurity and school outcomes. Paediatr Perinat Epidemiol 14, 324–331 (2000). 11101019 10.1046/j.1365-3016.2000.00276.x

[b5] Volpe J. J. Encephalopathy of prematurity includes neuronal abnormalities. Pediatrics 116, 221–225, doi: 10.1542/peds (2005). 15995055

[b6] Volpe J. J. Brain injury in premature infants: a complex amalgam of destructive and developmental disturbances. Lancet Neurol 8, 110–124, doi: S1474-4422(08)70294-1 (2009). 19081519 10.1016/S1474-4422(08)70294-1 PMC2707149

[b7] Pandit A. S., Ball G., Edwards A. D. & Counsell S. J. Diffusion magnetic resonance imaging in preterm brain injury. Neuroradiology 55 Suppl 2, 65–95, doi: 10.1007/s00234-013-1242-x (2013). 23942765

[b8] Brown C. J. . Structural network analysis of brain development in young preterm neonates. Neuroimage 101, 667–680, doi: 10.1016/j.neuroimage (2014). 25076107

[b9] Kersbergen K. J. . Microstructural brain development between 30 and 40 weeks corrected age in a longitudinal cohort of extremely preterm infants. Neuroimage 103, 214–224, doi: 10.1016/j.neuroimage (2014). 25261000

[b10] Mathew P. . Maturation of corpus callosum anterior midbody is associated with neonatal motor function in eight preterm-born infants. Neural plasticity 2013, 359532, doi: 10.1155/2013/359532 (2013). 23509639 PMC3569930

[b11] Doria V. . Emergence of resting state networks in the preterm human brain. Proc. Natl. Acad. Sci. USA 107, 20015–20020, doi: 10.1073/pnas (2010). 21041625 PMC2993415

[b12] Smyser C. D. . Longitudinal analysis of neural network development in preterm infants. Cereb Cortex 20, 2852–2862, doi: 10.1093/cercor (2010). 20237243 PMC2978240

[b13] Constable R. T. . A left cerebellar pathway mediates language in prematurely-born young adults. Neuroimage 64, 371–378 (2013). 22982585 10.1016/j.neuroimage.2012.09.008 PMC3508203

[b14] Kwon S. H. . Functional magnetic resonance connectivity studies in infants born preterm: suggestions of proximate and long-lasting changes in language organization. Dev Med Child Neurol 58 Suppl 4, 28–34, doi: 10.1111/dmcn.13043 (2016). 27027605 PMC6426123

[b15] Dennis E. L. & Thompson P. M. Typical and atypical brain development: a review of neuroimaging studies. Dialogues in clinical neuroscience 15, 359–384 (2013). 24174907 10.31887/DCNS.2013.15.3/edennis PMC3811107

[b16] Uddin L., Supekar K. & Menon V. Typical and atypical development of functional human brain networks: insights from resting-state fMRI. Frontiers in Systems Neuroscience (2010). 10.3389/fnsys.2010.00021 PMC2889680 20577585

[b17] Barnea-Goraly N. . White matter tract alterations in fragile X syndrome: preliminary evidence from diffusion tensor imaging. American journal of medical genetics. Part B, Neuropsychiatric genetics : the official publication of the International Society of Psychiatric Genetics 118, 81–88 (2003). 10.1002/ajmg.b.10035 12627472

[b18] Haas B. W. . Early white-matter abnormalities of the ventral frontostriatal pathway in fragile X syndrome. Developmental medicine and child neurology 51, 593–599, doi: 10.1111/j.1469 (2009). 19416325 PMC2715437

[b19] Haas B. W. . Preliminary evidence of abnormal white matter related to the fusiform gyrus in Williams syndrome: a diffusion tensor imaging tractography study. Genes, brain, and behavior 11, 62–68, doi: 10.1111/gbb (2012). PMC5575913 21939500

[b20] Maximo J. O., Cadena E. J. & Kana R. K. The implications of brain connectivity in the neuropsychology of autism. Neuropsychology review 24, 16–31, doi: 10.1007/s11065 (2014). 24496901 PMC4059500

[b21] Pavuluri M. N. . Diffusion tensor imaging study of white matter fiber tracts in pediatric bipolar disorder and attention-deficit/hyperactivity disorder. Biol Psychiatry 65, 586–593, doi: 10.1016/j.biopsych (2009). 19027102 PMC2677389

[b22] Rane P. . Connectivity in Autism: A Review of MRI Connectivity Studies. Harvard review of psychiatry 23, 223–244, doi: 10.1097/hrp (2015). 26146755 PMC5083037

[b23] Ray S. . Structural and functional connectivity of the human brain in autism spectrum disorders and attention-deficit/hyperactivity disorder: A rich club-organization study. Hum Brain Mapp 35, 6032–6048, doi: 10.1002/hbm.22603 (2014). 25116862 PMC4319550

[b24] Sripada C. . Disrupted network architecture of the resting brain in attention-deficit/hyperactivity disorder. Hum Brain Mapp 35, 4693–4705, doi: 10.1002/hbm.22504 (2014). 24668728 PMC6869736

[b25] Uddin L. Q., Supekar K. & Menon V. Reconceptualizing functional brain connectivity in autism from a developmental perspective. Front Hum Neurosci 7, 458, doi: 10.3389/fnhum.2013.00458 (2013). 23966925 PMC3735986

[b26] von Rhein D. . Aberrant local striatal functional connectivity in attention-deficit/hyperactivity disorder. J Child Psychol Psychiatry, doi: 10.1111/jcpp.12529 (2016). 26871610

[b27] Wang J. Y., Hessl D. H., Hagerman R. J., Tassone F. & Rivera S. M. Age-dependent structural connectivity effects in fragile x premutation. Arch Neurol 69, 482–489, doi: 10.1001/archneurol.2011.2023 (2012). 22491193 PMC3979438

[b28] Debbane M. . Resting-state networks in adolescents with 22q11.2 deletion syndrome: associations with prodromal symptoms and executive functions. Schizophr Res 139, 33–39, doi: 10.1016/j.schres.2012.05.021 (2012). 22704643

[b29] Hoeft F. . More is not always better: increased fractional anisotropy of superior longitudinal fasciculus associated with poor visuospatial abilities in Williams syndrome. J Neurosci 27, 11960–11965, doi: 10.1523/jneurosci.3591-07.2007 (2007). 17978036 PMC6673356

[b30] Koyama M. S. . Cortical signatures of dyslexia and remediation: an intrinsic functional connectivity approach. PLoS One 8, e55454, doi: 10.1371/journal.pone.0055454 (2013). 23408984 PMC3569450

[b31] Mankinen K. . Connectivity disruptions in resting-state functional brain networks in children with temporal lobe epilepsy. Epilepsy research 100, 168–178, doi: 10.1016/j.eplepsyres.2012.02.010 (2012). 22418271

[b32] Molko N. . Functional and structural alterations of the intraparietal sulcus in a developmental dyscalculia of genetic origin. Neuron 40, 847–858 (2003). 14622587 10.1016/s0896-6273(03)00670-6

[b33] Molko N. . Brain anatomy in Turner syndrome: evidence for impaired social and spatial-numerical networks. Cereb Cortex 14, 840–850, doi: 10.1093/cercor/bhh042 (2004). 15054057

[b34] Papadelis C. . Cortical somatosensory reorganization in children with spastic cerebral palsy: a multimodal neuroimaging study. Front Hum Neurosci 8, 725, doi: 10.3389/fnhum.2014.00725 (2014). 25309398 PMC4162364

[b35] Pujol J. . Anomalous brain functional connectivity contributing to poor adaptive behavior in Down syndrome. Cortex; a journal devoted to the study of the nervous system and behavior 64, 148–156, doi: 10.1016/j.cortex.2014.10.012 (2015). 25461715

[b36] Vadivelu S., Wolf V. L., Bollo R. J., Wilfong A. & Curry D. J. Resting-state functional MRI in pediatric epilepsy surgery. Pediatric neurosurgery 49, 261–273, doi: 10.1159/000363605 (2013). 25277135

[b37] Vega J. N., Hohman T. J., Pryweller J. R., Dykens E. M. & Thornton-Wells T. A. Resting-State Functional Connectivity in Individuals with Down Syndrome and Williams Syndrome Compared with Typically Developing Controls. Brain Connect 5, 461–475, doi: 10.1089/brain.2014.0266 (2015). 25712025 PMC4601631

[b38] Lubsen J. . Microstructural and functional connectivity in the developing preterm brain. Semin Perinatol 35, 34–43, doi: 10.1053/j.semperi.2010.10.006 (2011). 21255705 PMC3063450

[b39] Roze E. . Neonatal DTI early after birth predicts motor outcome in preterm infants with periventricular hemorrhagic infarction. Pediatr Res 78, 298–303, doi: 10.1038/pr.2015.94 (2015). 25978802

[b40] Berger R. & Soder S. Neuroprotection in preterm infants. BioMed research international 2015, 257139, doi: 10.1155/2015/257139 (2015). 25650134 PMC4306255

[b41] Elitt C. M. & Rosenberg P. A. The challenge of understanding cerebral white matter injury in the premature infant. Neuroscience 276, 216–238, doi: 10.1016/j.neuroscience.2014.04.038 (2014). 24838063 PMC4146717

[b42] Loeliger M. . High-frequency oscillatory ventilation is not associated with increased risk of neuropathology compared with positive pressure ventilation: a preterm primate model. Pediatr Res 66, 545–550, doi: 10.1203/PDR.0b013e3181bb0cc1 (2009). 19687780 PMC2804748

[b43] Pineda R. G. . Alterations in brain structure and neurodevelopmental outcome in preterm infants hospitalized in different neonatal intensive care unit environments. J Pediatr 164, 52–60.e52, doi: 10.1016/j.jpeds.2013.08.047 (2014). 24139564 PMC3872171

[b44] Thomason M. E. . Cross-hemispheric functional connectivity in the human fetal brain. Science translational medicine 5, doi: 10.1126/scitranslmed.3004978 (2013). PMC3618956 23427244

[b45] Schopf V., Kasprian G., Brugger P. C. & Prayer D. Watching the fetal brain at ‘rest’. Int J Dev Neurosci 30, 11–17, doi: 10.1016/j.ijdevneu.2011.10.006 (2012). 22044604

[b46] Scheinost D. . The intrinsic connectivity distribution: a novel contrast measure reflecting voxel level functional connectivity. Neuroimage 62, 1510–1519, doi: 10.1016/j.neuroimage.2012.05.073 (2012). 22659477 PMC3538880

[b47] Batalle D. . Altered small-world topology of structural brain networks in infants with intrauterine growth restriction and its association with later neurodevelopmental outcome. Neuroimage 60, 1352–1366, doi: 10.1016/j.neuroimage.2012.01.059 (2012). 22281673

[b48] DiPietro J. A. & Voegtline K. M. The gestational foundation of sex differences in development and vulnerability. Neuroscience, doi: 10.1016/j.neuroscience.2015.07.068 (2015). PMC4732938 26232714

[b49] Buss C., Davis E. P., Muftuler L. T., Head K. & Sandman C. A. High pregnancy anxiety during mid-gestation is associated with decreased gray matter density in 6–9-year-old children. Psychoneuroendocrinology 35, 141–153, doi: 10.1016/j.psyneuen.2009.07.010 (2010). 19674845 PMC2795128

[b50] Hackman D. A. & Farah M. J. Socioeconomic status and the developing brain. Trends in cognitive sciences 13, 65–73, doi: 10.1016/j.tics.2008.11.003 (2009). 19135405 PMC3575682

[b51] Kwon S. H. . Adaptive mechanisms of developing brain: Cerebral lateralization in the prematurely-born. Neuroimage 108C, 144–150, doi: 10.1016/j.neuroimage.2014.12.032 (2014). PMC4324328 25528658

[b52] Burd I. . Beyond white matter damage: fetal neuronal injury in a mouse model of preterm birth. Am J Obstet Gynecol 201, 279.e271-278, doi: 10.1016/j.ajog.2009.06.013 (2009). 19733279 PMC2740757

[b53] Wilke M., Hauser T. K., Krageloh-Mann I. & Lidzba K. Specific impairment of functional connectivity between language regions in former early preterms. Hum Brain Mapp 35, 3372–3384, doi: 10.1002/hbm.22408 (2014). 24243552 PMC6869459

[b54] Scheinost D. . Cerebral Lateralization is Protective in the Very Prematurely Born. Cereb Cortex, 25, 1858–66, doi: 10.1093/cercor/bht430 (2015). 24451659 PMC4459290

[b55] Gozzo Y. . Alterations in neural connectivity in preterm children at school age. NeuroImage 48, 458–463 (2009). 19560547 10.1016/j.neuroimage.2009.06.046 PMC2775072

[b56] Luu T. M., Vohr B. R., Allan W., Schneider K. C. & Ment L. R. Evidence for catch-up in cognition and receptive vocabulary among adolescents born very preterm. Pediatrics 128, 313–322, doi: 10.1542/peds.2010-2655 (2011). 21768322 PMC3146356

[b57] Thomason M. E. . Intrinsic functional brain architecture derived from graph theoretical analysis in the human fetus. PLoS One (2014). 10.1371/journal.pone.0094423 PMC4006774 24788455

[b58] Yoon B. H. . Amniotic fluid inflammatory cytokines (interleukin-6, interleukin-1beta, and tumor necrosis factor-alpha), neonatal brain white matter lesions, and cerebral palsy. Am J Obstet Gynecol 177, 19–26 (1997). 9240577 10.1016/s0002-9378(97)70432-0

[b59] Yoon B. H. . Experimentally induced intrauterine infection causes fetal brain white matter lesions in rabbits. Am J Obstet Gynecol 177, 797–802 (1997). 9369822 10.1016/s0002-9378(97)70271-0

[b60] Smyser C. D. . Effects of white matter injury on resting state fMRI measures in prematurely born infants. PLoS One 8, e68098, doi: 10.1371/journal.pone.0068098 (2013). 23874510 PMC3706620

[b61] Cao Q. . Abnormal neural activity in children with attention deficit hyperactivity disorder: a resting-state functional magnetic resonance imaging study. Neuroreport 17, 1033–1036 (2006). 16791098 10.1097/01.wnr.0000224769.92454.5d

[b62] Noonan S. K., Haist F. & Muller R. A. Aberrant functional connectivity in autism: evidence from low-frequency BOLD signal fluctuations. Brain Res 1262, 48–63, doi: 10.1016/j.brainres.2008.12.076 (2009). 19401185 PMC2766184

[b63] Tian L. . Altered resting-state functional connectivity patterns of anterior cingulate cortex in adolescents with attention deficit hyperactivity disorder. Neurosci Lett 400, 39–43 (2006). 16510242 10.1016/j.neulet.2006.02.022

[b64] Weng S. J. . Alterations of resting state functional connectivity in the default network in adolescents with autism spectrum disorders. Brain Res 1313, 202–214, doi: 10.1016/j.brainres.2009.11.057 (2010). 20004180 PMC2818723

[b65] Blankenship A. G. & Feller M. B. Mechanisms underlying spontaneous patterned activity in developing neural circuits. Nat Rev Neurosci 11, 18–29, doi: 10.1038/nrn2759 (2010). 19953103 PMC2902252

[b66] Tovar-Moll F. . Structural and functional brain rewiring clarifies preserved interhemispheric transfer in humans born without the corpus callosum. Proc Natl Acad Sci USA 111, 7843–7848, doi: 10.1073/pnas.1400806111 (2014). 24821757 PMC4040546

[b67] Hack M. . Poor predictive validity of the Bayley Scales of Infant Development for cognitive function of extremely low birth weight children at school age. Pediatrics 116, 333–341, doi: 10.1542/peds.2005-0173 (2005). 16061586

[b68] Ment L. R. . Change in cognitive function over time in very low-birth-weight infants. Jama 289, 705–711 (2003). 12585948 10.1001/jama.289.6.705

[b69] Markham R. G., Shimizu T. & Lickliter R. Extrinsic embryonic sensory stimulation alters multimodal behavior and cellular activation. Developmental neurobiology 68, 1463–1473, doi: 10.1002/dneu.20667 (2008). 18777564 PMC2647013

[b70] Van den Heuvel M. I. & Thomason M. E. Functional Connectivity of the Human Brain in Utero. Trends Cogn Sci. 20(12), 931–939, doi: 10.1016/j.tics.2016.10.001 (2016). 27825537 PMC5339022

[b71] Partanen E. . Learning-induced neural plasticity of speech processing before birth. Proc Natl Acad Sci USA 110, 15145–15150, doi: 10.1073/pnas.1302159110 (2013). 23980148 PMC3773755

[b72] Glover G. H., Li T. Q. & Ress D. Image-based method for retrospective correction of physiological motion effects in fMRI: RETROICOR. Magn Reson Med 44, 162–167 (2000). 10893535 10.1002/1522-2594(200007)44:1<162::aid-mrm23>3.0.co;2-e

[b73] Kochiyama T. . Removing the effects of task-related motion using independent-component analysis. Neuroimage 25, 802–814, doi: 10.1016/j.neuroimage.2004.12.027 (2005). 15808981

[b74] Avants B. B. . The Insight ToolKit image registration framework. Front Neuroinform 8, 44, doi: 10.3389/fninf.2014.00044 (2014). 24817849 PMC4009425

[b75] Power J. D., Barnes K. A., Snyder A. Z., Schlaggar B. L. & Petersen S. E. Spurious but systematic correlations in functional connectivity MRI networks arise from subject motion. Neuroimage 59, 2142–2154, doi: 10.1016/j.neuroimage.2011.10.018 (2012). 22019881 PMC3254728

[b76] Serag A. . Construction of a consistent high-definition spatio-temporal atlas of the developing brain using adaptive kernel regression. Neuroimage 59, 2255–2265, doi: 10.1016/j.neuroimage.2011.09.062 (2012). 21985910

[b77] Calhoun V. D., Adali T., Pearlson G. D. & Pekar J. J. A method for making group inferences from functional MRI data using independent component analysis. Human Brain Mapping 14, 140–151, doi: 10.1002/hbm.1048 (2001). 11559959 PMC6871952

[b78] Craddock R. C., James G. A., Holtzheimer P. E. 3rd, Hu X. P. & Mayberg H. S. A whole brain fMRI atlas generated via spatially constrained spectral clustering. Hum Brain Mapp 33, 1914–1928, doi: 10.1002/hbm.21333 (2012). 21769991 PMC3838923

[b79] Shehzad Z. . A multivariate distance-based analytic framework for connectome-wide association studies. Neuroimage 93 Pt 1, 74–94, doi: 10.1016/j.neuroimage.2014.02.024 (2014). 24583255 PMC4138049

[b80] Logothetis N. K., Pauls J., Augath M., Trinath T. & Oeltermann A. Neurophysiological investigation of the basis of the fMRI signal. Nature 412, 150–157, doi: 10.1038/35084005 (2001). 11449264

[b81] Thomason M. E. . Age-related increases in long-range connectivity in fetal functional neural connectivity networks in utero. Developmental cognitive neuroscience 11, 96–104, doi: 10.1016/j.dcn.2014.09.001 (2015). 25284273 PMC4532276

[b82] Jakab A. . Fetal functional imaging portrays heterogeneous development of emerging human brain networks. Front Hum Neurosci 8, 852, doi: 10.3389/fnhum.2014.00852 (2014). 25374531 PMC4205819

[b83] Kostović I. & Jovanov-Milosevic N. The development of cerebral connections during the first 20–45 weeks’ gestation. Semin Fetal Neonatal Med 11, 415–422, doi: 10.1016/j.siny.2006.07.001(2006). 16962836

[b84] Yakovlev P. I. & Lecours A. R. In Regional Development of the Brain in Early Life (ed. Minkowski A.) 3–70 (Blackwell, 1967).

[b85] Arichi T. . Development of BOLD signal hemodynamic responses in the human brain. Neuroimage 63, 663–673 (2012). 22776460 10.1016/j.neuroimage.2012.06.054 PMC3459097

[b86] Colonnese M. T., Phillips M. A., Constantine-Paton M., Kaila K. & Jasanoff A. Development of hemodynamic responses and functional connectivity in rat somatosensory cortex. Nat Neurosci 11, 72–79, doi: 10.1038/nn2017 (2008). 18037883

[b87] Conradi N. G., Engvall J. & Wolff J. R. Angioarchitectonics of rat cerebellar cortex during pre- and postnatal development. Acta Neuropathol 50, 131–138 (1980). 7395467 10.1007/BF00692863

[b88] Joshi A. . Unified framework for development, deployment and robust testing of neuroimaging algorithms. Neuroinformatics 9, 69–84, doi: 10.1007/s12021-010-9092-8 (2011). 21249532 PMC3066099

[b89] Mitchell M. R. . A preliminary investigation of Stroop-related intrinsic connectivity in cocaine dependence: associations with treatment outcomes. Am J Drug Alcohol Abuse 39, 392–402, doi: 10.3109/00952990.2013.841711 (2013). 24200209 PMC3827911

