# Supplementary material for: Weak functional connectivity in the human fetal brain prior to preterm birth
Source: Sci Rep. 2017 Jan 9;7:39286. doi: 10.1038/srep39286 (PMC5221666; doi:10.1038/srep39286)
Supplement: Supplementary Information [file srep39286-s1.doc]

Weak functional connectivity in the human fetal brain prior to preterm birth

Authors: Moriah E. Thomason, Dustin Scheinost, Janessa H. Manning, Lauren E. Grove, Jasmine Hect, Narcis Marshall, Edgar Hernandez-Andrade, Susan Berman, Athina Pappas, Lami Yeo, Sonia S. Hassan, R. Todd Constable, Laura R. Ment, Roberto Romero

**Supplementary Material**

**
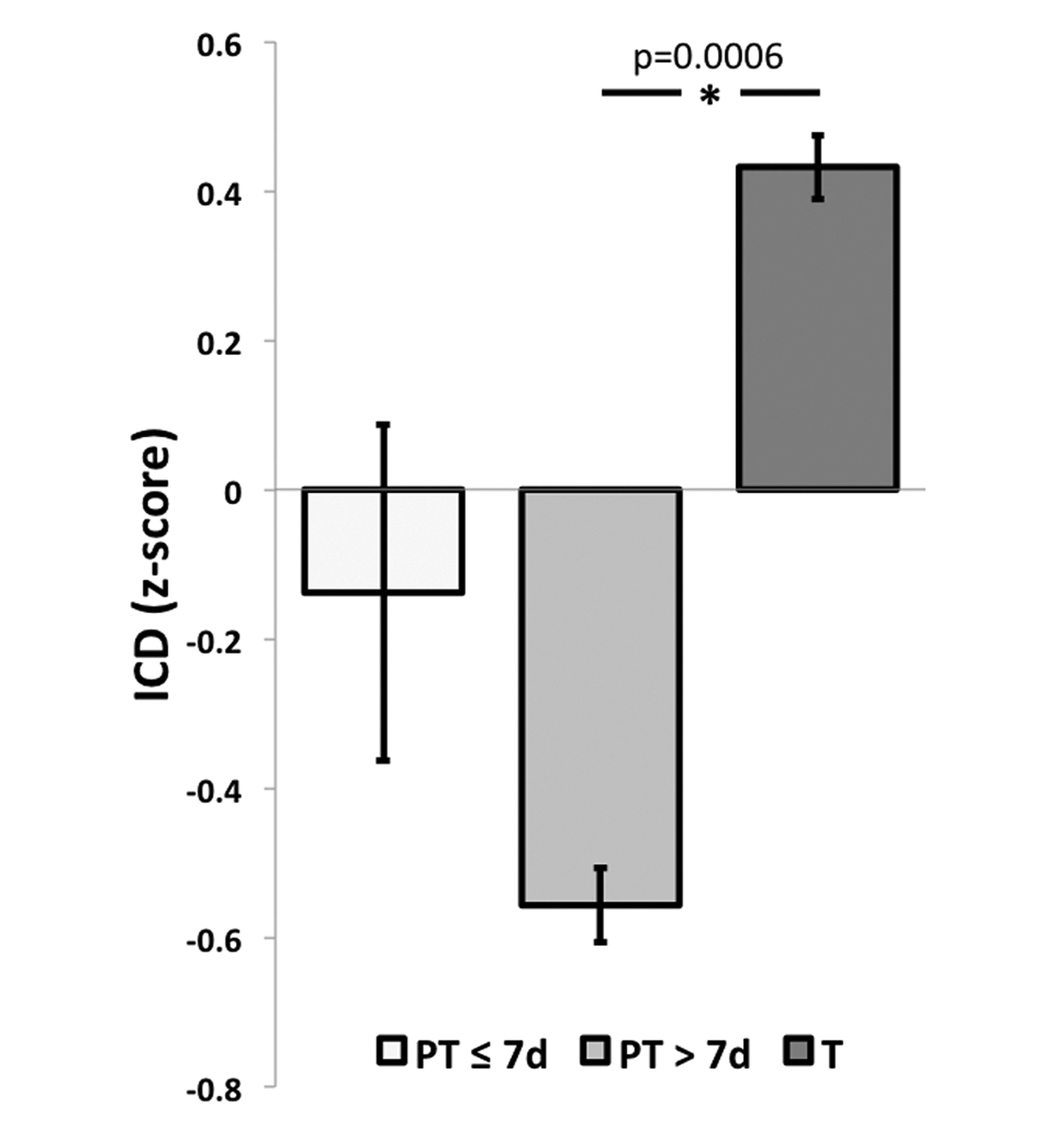
**

**Supplemental Figure 1:** Evaluation of whether onset of labor contributed to observed effects. While on average the difference between the day of scan and day of birth for the preterm-born fetuses was 19.3 days, four fetuses were born within 7 days of the fMRI scan. Using a region of interest covering all significant voxels in Figure 1C, we compared average ICD values for fetuses born preterm within 7 days of the scan, fetuses born preterm more than 7 days after the scan, and term-born fetuses. Comparisons between preterm-born fetuses born more than 7 days after the scan, and term fetuses remained significant (t=3.89, p<0.001, df=26), suggesting that the observed connectivity differences were not related to possible labor effects. **PT – preterm born fetuses; T – term born fetuses.**

**
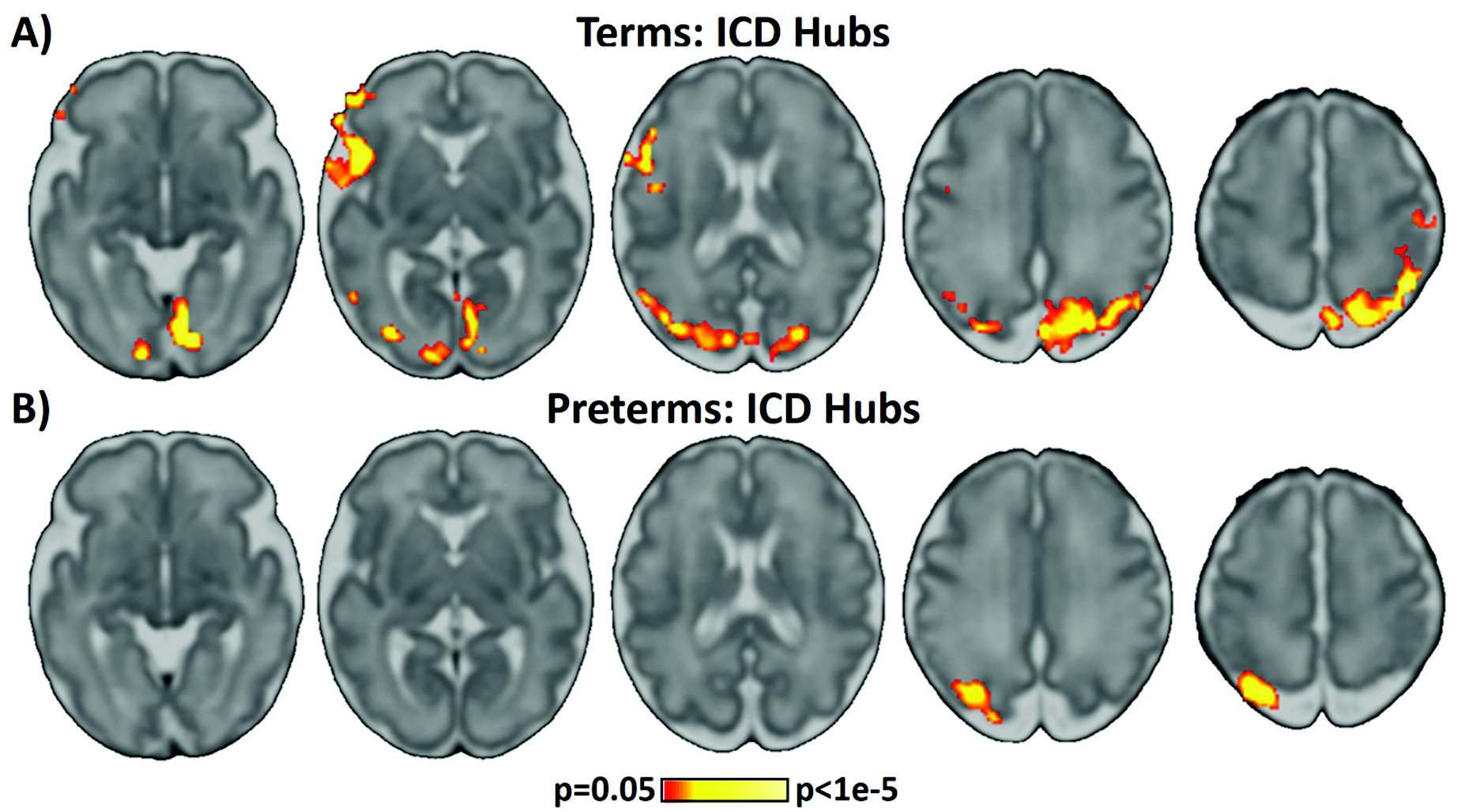
**

**Supplemental Figure 2:** Axial slices of putative hubs of the fetal connectome. ICD hubs were defined as regions with significantly greater (p<0.05, corrected, one-tailed) ICD values relative to the whole brain average ICD value. A) Term-born fetuses exhibited a high degree of connectivity in posterior brain regions extending into midline posterior cingulate cortex, and in left hemisphere prefrontal regions that may later support expressive language. B) In contrast, preterm-born fetuses only exhibited a high degree of connectivity in the posterior parietal lobe.

**
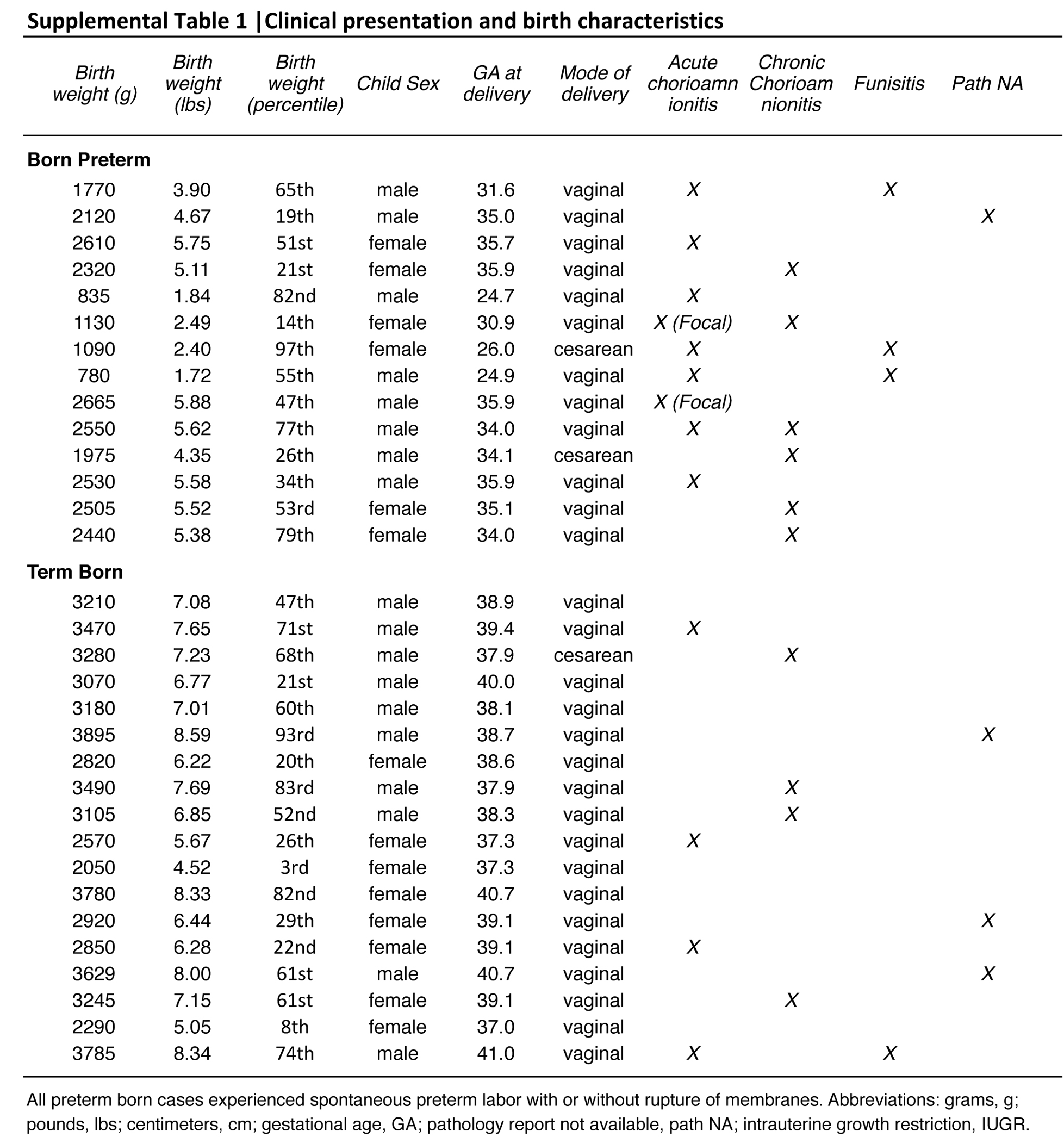
**

**
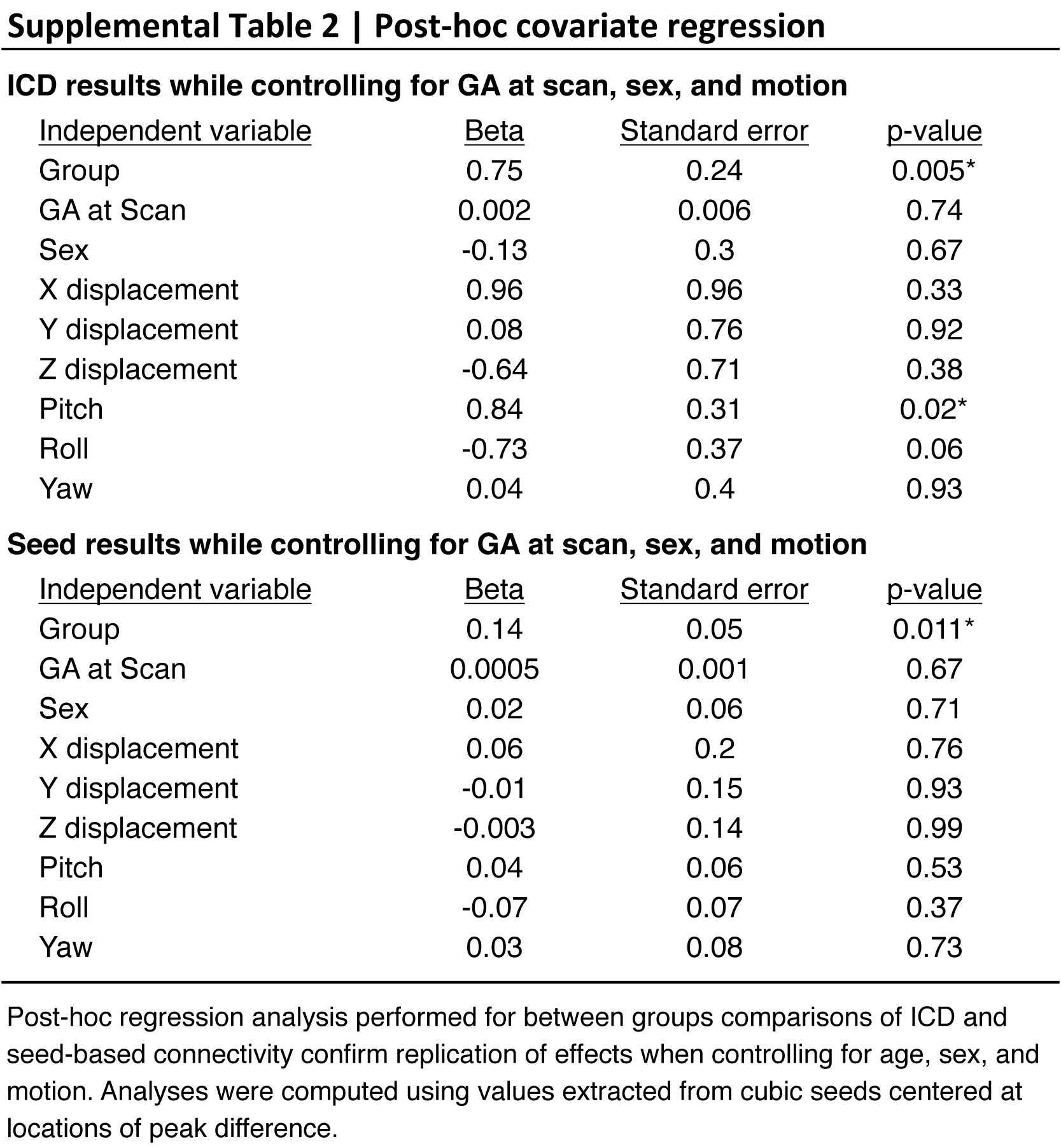
**
